# Alcohol abuse as the strongest risk factor for violent offending in patients with paranoid schizophrenia

**DOI:** 10.3325/cmj.2014.55.156

**Published:** 2014-04

**Authors:** Marija Kudumija Slijepčević, Vlado Jukić, Darko Novalić, Tija Žarković-Palijan, Milan Milošević, Ivana Rosenzweig

**Affiliations:** 1 Department of Psychiatry, General Hospital Bjelovar, Bjelovar, Croatia; 2 Technical College Bjelovar, Bjelovar, Croatia; 3 Psychiatric Clinical Hospital Vrapče, Zagreb, Croatia; 4 Psychiatric Hospital “Dr Ivan Barbot,” Popovača, Croatia; 5 Department for Environmental and Occupational Health, University of Zagreb, School of Medicine, Andrija Štampar School of Public Health, Zagreb, Croatia; 6 Department of Neuroimaging, Institute of Psychiatry, King's College London, London, United Kingdom; 7 Danish Epilepsy Centre, Dianalund, Denmark

## Abstract

**Aim:**

To determine predictive risk factors for violent offending in patients with paranoid schizophrenia in Croatia.

**Method:**

The cross-sectional study including male in-patients with paranoid schizophrenia with (N = 104) and without (N = 102) history of physical violence and violent offending was conducted simultaneously in several hospitals in Croatia during one-year period (2010-2011). Data on their sociodemographic characteristics, duration of untreated illness phase (DUP), alcohol abuse, suicidal behavior, personality features, and insight into illness were collected and compared between the groups. Binary logistic regression model was used to determine the predictors of violent offending.

**Results:**

Predictors of violent offending were older age, DUP before first contact with psychiatric services, and alcohol abuse. Regression model showed that the strongest positive predictive factor was harmful alcohol use, as determined by AUDIT test (odds ratio 37.01; 95% confidence interval 5.20-263.24). Psychopathy, emotional stability, and conscientiousness were significant positive predictive factors, while extroversion, pleasantness, and intellect were significant negative predictive factors for violent offending.

**Conclusion:**

This study found an association between alcohol abuse and the risk for violent offending in paranoid schizophrenia. We hope that this finding will help improve public and mental health prevention strategies in this vulnerable patient group.

Individuals with schizophrenia have an increased risk of violence (1), but different studies report different risks (1,2). Anglo-American studies commonly report higher prevalence rates than European studies (3,4). These patients have also been reported to have up to 4-6 times higher violent behavior rate than the general population (3-5). Nonetheless, less than 0.2% patients suffering from schizophrenia commit homicide (in 20-year period) and less than 10% of commit a violent act (3). Also, patients with schizophrenia contribute to 6%-11% of all homicides and homicide attempts (3-5).

In general, aggressiveness is usually associated with anti-social personality features, juvenile delinquency, and psychoactive substance abuse (6). In patients with schizophrenia violence and violent offending is associated with a great number of risk factors, such as premorbid affinity to violent behavior, alcohol abuse, younger age, lower socioeconomic status (6,7), deinstitutionalization, longer duration of untreated psychosis, later onset of first episode of psychosis (1,4,8), lower social status, broken families, asocial behavior of parents, loss of father at an early age, a new marriage partner in the family, and growing up in an orphanage (9).

Several studies (10-12) looked at four basic personality dimensions and their role in violence in patients with schizophrenic illness spectrum: impulse control, affect regulation, narcissism, and paranoid cognition. Impulsivity and immature affect regulation were associated with most neuropsychiatric disorders, and were particularly predictive of affinity for addictive disorders, while paranoid cognition and narcissism were predictive of violence acts (10-12).

The causes of schizophrenia may be genetic, early environmental, and epigenetic risk factors (13,14), which may further modulate the risk of violent offending among individuals with this disease (1,15). Until recently, very little has been reported about the predictive factors of violence and violent offending in the patient population in Croatia. The Croatian population has during the last two decades been exposed to environmental and socio-demographic changes (eg, Croatian War 1991-1995 and post-war period), which might have had an impact on predictive risk factors. Therefore, we conducted a cross-sectional study of in-patients with paranoid schizophrenia with or without history of physical violence and violent offending (inclusive of homicide) in several hospitals in Croatia during one-year period.

## Patients and methods

### Patients and their sociodemographics

The study took place between December 1, 2010 and December 1, 2011. It included 206 male adult inpatients aged between 18 and 60 years who met the criteria for paranoid schizophrenia according to the fourth edition of the Diagnostic and Statistical Manual of Mental Disorders (DSM-IV) (16) and who agreed to be part of the study. Non-inclusion criteria included neurological or significant somatic illnesses, learning disability, or organic brain damage.

Of these patients, 102 (non-violent offending) were voluntary inpatients at two acute psychiatric departments (General Hospital Bjelovar and Psychiatric Clinical Hospital Vrapče) with no recorded or reported history of violent offending. Open Aggressiveness Scale (OAS) (17) was administered at the admission and the cut-off point of 7 was established as the inclusion criterion (18).

Hundred and four male adult patients (violent offending group) were involuntary inpatients, hospitalized at forensic departments (Psychiatric Clinical Hospital Vrapče and Psychiatric Hospital “Dr Ivan Barbot” in Popovača) following the incidence of violence and violent offending, inclusive of homicide or attempted homicide. Twenty four patients left the study during assessments: 6 from non-violent offending group – due to inability to concentrate and 18 from violent offending group – 3 due to inability to concentrate, 4 due to sight impairment, 3 due to the fear that the results would be used against them, and 8 offered no explanation. Written informed consent was obtained from all participants and research was approved by the respective institutional ethics committees.

### Methods

To insure consistency and intra- and inter-rater reliability, all the clinical assessments, data collection, and questionnaire application were carried out by a group of researchers and specialized psychiatrists. They were all trained for the purposes of this study and were tested for consistency for the used scales and DSM-IV criteria application for the diagnosis of paranoid schizophrenia (16).

Clinical assessment involved the following: structured psychiatric interview, collection of socio-demographic data (using a semi-structured questionnaire), duration of untreated psychosis (DUP) assessment (objective and subjective), AUDIT questionnaire application (19), and InterSePT questionnaire application (20).

The following general and socio-demographic data were collected: age, age of the primary contact with psychiatric services, early psychomotor development history, family cohesion, parents’ education, patient’s educational, social, and employment status (inclusive of salary/pension level), marital status and the number of children, urbanicity of the place of residence and rural-urban migration, family psychiatric and medical history, and asocial behavior in the primary family.

For the assessment of alcohol consumption we used the AUDIT questionnaire, which covers three separable domains: consumption, harmful use, and dependent use (19). The score of 8 and more was used as the limit for determining harmful alcohol use (21). For the assessment of current suicidal ideation we used 12-item InterSePTScale for Suicidal Thinking (20). DUP was assessed by clinical interview and the recorded psychiatric history in medical files. The presence of three groups of symptoms was determined – positive symptoms (hallucinations, delusions, and odd beliefs thought disorder), negative symptoms (depression, dysphoria, apathy, anergia, apathy, and amotivation), and signs of social decline (withdrawn behavior, poor interpersonal relationship, social avoidance, and lack of interest in education or work). The patients also filled in the International Personality Item Pool (IPIP, *http://ipip.ori.org/ipip/*) self-assessment questionnaires, Brief Cognitive Insight Scale (BCIS) (22), and they self-assessed how mentally unwell they had been before their first psychiatric treatment (DUP self-assessment). Psychopathy was defined as the sum score of all IPIP items that described sociability, assertiveness, emotional reactivity, irritability, impoliteness, need to control, and irresponsibility (23).

### Statistical analysis

Standard descriptive statistics measures (mean, standard deviation [SD], medians with interquartile ranges) were used. Normality of distribution was tested by Smirnov-Kolmogorov test, and suitable nonparametric methods were applied. Differences in quantitative values between the two groups were tested by a nonparametric Mann-Whitney U test, while differences in categorical variables were tested by X^2^ test. Binary logistic regression was performed to assess the impact of several predictor variables (age, education, AUDIT score, InterSept score, Beck’s Cognitive Insight scale, psychopathy index, extraversion, agreeableness, consciousness, intellect, self-reflection, and duration of untreated psychosis) on the likelihood that patients had aggressive behavior. *P* values lower than or equal to 0.05 were considered significant. STATISTICA version 9.1 was used (*www.statsoft.com*).

## Results

### General characteristics

Violent offending group was significantly older (43.80 years vs 34.83 years), but there was no significant difference in age of first contact with psychiatric services (violent offending = 27.69 years, non-violent offending = 24.23 years, *P* > 0.05, χ^2^ test). Violent offending participants were five times more frequently children of divorced parents (84.5% vs 15.5%, *P* = 0.022, χ^2^ test) and significantly fewer of them had finished high school (13.5% non-violent offending vs 0% violent offending participants, *P* < 0.001). Although there were no significant differences in school grades (the most common grades were good and very good in both groups, over 75% of participants), significantly more violent offending participants lost a year (*P* = 0.001, χ^2^ test). Non-violent offending participants had significantly higher incomes (18.2% vs 1.2%, *P* < 0.001, χ^2^ test) and were four times more likely to be married (Table 1).

**Table 1:** General patients' characteristics (χ^2^ test)

		Group				*P*
		Non-violent offending, N = 96		Violent offending, N = 86		
		No.	%	No.	%	
Education	without school	0	0.00	2	2.33	<0.001
	elementary	17	17.71	25	29.07	
	high school	66	68.75	53	61.63	
	college	0	0.00	6	6.98	
	university	13	13.54	0	0.00	
Marriage status	no	75	78.13	76	93.83	0.003
	yes	21	21.88	5	6.17	
Significant somatic co-morbidity and/or history	no	81	84.38	71	82.56	0.742
	yes	15	15.63	15	17.44	
Head injury	no	63	65.63	45	52.94	0.083
	yes	33	34.38	40	47.06	
Urbanicity	no	26	27.08	48	56.47	<0.001
	yes	70	72.92	37	43.53	
Divorced parents	no	91	94.79	71	84.52	0.022
	yes	5	5.21	13	15.48	
Problems with the law	no	76	79.17	75	88.24	0.102
	yes	20	20.83	10	11.76	
Monthly income (HRK)	<1000	30	34.09	37	44.05	0.001
	1000-3000	29	32.95	38	45.24	
	3000-5000	13	14.77	8	9.52	
	>5000	16	18.18	1	1.19	
Positive psychiatric family history	no	76	79.17	65	76.47	0.663
	yes	20	20.83	20	23.53	
Age, median (IQR)		34.00 (26.00-43.75)		43.50 (36.00-52.25)		<0.001*
Duration of untreated psychosis (months), median (IQR)		8.00 (3.00-13.50)		12.00 (6.00-24.00)		<0.001*
AUDIT score, median (IQR)		0.00 (0.00-4.00)		2.00 (0.00-8.00)		0.072*

*Mann-Whitney U test.

### Duration of the untreated illness phase

Both groups showed distorted insight as to when their psychosis had started and how long it had lasted (DUP self-assessment). The mean self-assessed DUP was 7.16 months in violent offending participants (SD 12.08; median 0.00 months) and 6.44 months in non-violent offending participants (SD 7.05, median 3.00 months, *P* = 0.024, Mann-Whitney test). Violent offending participants had significantly longer period of psychiatric assessment – 14.33 months (SD 9.20; median 12.00 months) than non-violent offending participants (9.45 months, SD 6.95; median 8.00 months, *P* < 0.001, Mann-Whitney Test).

### Suicidal behavior, personality traits, insight into the illness, and alcohol abuse

No significant difference between the groups was found in suicidal thoughts and behavior (*P* = 0.176, Mann-Whitney test). InterSePT scale (20) had a satisfactory internal consistency (Cronbach α = 0.82). Non-violent offending group had a significantly higher extroversion score – 31.58 (SD 7.47; median 15.00) than violent offending group (27.093, SD 10.73; median 24.00, *P* = 0.001, Mann-Whitney U test) (Table 2). Violent offending participants had a significantly higher mean conscientiousness score – 38.79 (SD 6.88; median 39.00, *P* = 0.038, Mann-Whitney U test) and significantly higher mean emotional stability score – 33.21 (SD 9.72; median 34.50, *P* = 0.011, Mann-Whitney U test). No significant differences were found between the groups in psychopathy index (*P* = 0.110, Mann-Whitney U test) and in insight into the illness (Table 2). BCIS scale had a satisfactory internal consistency (Cronbach α = 0.84). Violent offending group was almost nine times more likely to be addicted to alcohol (AUDIT score >8; 27.1% vs 3.1%, *P* < 0.001, χ^2^ test).

**Table 2:** Differences in individual questionnaire items and Beck's Cognitive Insight Scale (BCIS) subscales between violent offending and non-violent offending group (Mann-Whitney U-test)

	Group	N	Min	Max	Percentiles			*P*
					25th	Median	75th	
IPIP 50: Extraversion	violent offending	86	5.0	48.0	18.00	24.00	35.00	0.001
	non-violent offending	96	15.0	48.0	26.00	33.00	38.00	
IPIP 50: Agreeableness	violent offending	86	4.0	62.0	30.75	36.00	41.00	0.097
	non-violent offending	96	24.0	49.0	33.25	38.00	41.75	
IPIP 50: Consciousness	violent offending	86	3.0	50.0	36.00	39.00	44.00	0.038
	non-violent offending	96	19.0	48.0	34.00	37.00	41.00	
IPIP 50: Emotional stability	violent offending	86	3.0	50.0	27.75	34.50	40.25	0.011
	non-violent offending	96	16.0	44.0	26.00	29.00	37.00	
IPIP 50: Intellect	violent offending	86	3.0	50.0	26.00	33.00	38.25	0.111
	non-violent offending	96	19.0	50.0	31.00	34.00	39.75	
Self-reflectiveness subscale	violent offending	86	0.00	26.00	9.00	13.00	16.00	0.396
	non-violent offending	96	0.00	23.00	11.00	14.00	14.75	
Self-certainty subscale	violent offending	86	0.00	18.00	6.00	10.00	13.25	0.377
	non-violent offending	96	0.00	18.00	7.00	9.50	13.00	
BCIS composite index	violent offending	86	-12.00	15.00	0.00	2.50	6.00	0.218
	non-violent offending	96	-4.00	15.00	1.00	4.00	7.00	

### Predictors for belonging to the violent-offending

Binary logistic regression model was significant (X^2^_13_ test = 108.7, *P* < 0.001). It explained 61.3% of variance and correctly classified 85.0% of participants. Hosmer and Lemeshow test was not significant (*P* = 0.201). These data additionally indicate the validity of used regression model (Table 3).

**Table 3:** Predictors of belonging to the violent offending group: binary logistic regression

	Odds ratio	95% confidence interval	*P*
Age	1.08	1.03-1.12	0.001
Education	0.60	0.30-1.19	0.142
AUDIT>8	37.01	5.20-263.24	<0.001
Clinical impression (InterSePT)	0.92	0.46-1.85	0.807
Psychopathy index	6.23	1.16-33.32	0.033
Extraversion	0.90	0.83-0.97	0.009
Agreeableness	0.89	0.81-0.98	0.019
Consciousness	1.13	1.02-1.25	0.016
Emotional stability	1.19	1.09-1.29	<0.001
Intellect	0.90	0.82-0.99	0.035
Beck's Cognitive Insight Scale	0.97	0.85-1.11	0.669
Self-reflectiveness	1.06	0.92-1.21	0.432
Duration of untreated psychosis (months)	1.10	1.05-1.16	<0.001

Received: October 29, 2012

Accepted: February 13, 2014

The strongest positive violent offending predictor was AUDIT score higher than 8 (odds ratio [OR] 37.01, 95% confidence interval [CI] 5.20-263.24). These results suggest that the participants with the AUDIT score higher than 8 would have 37 times greater chances of belonging to violent offending group, with all other model variables controlled. Other significant positive violent offending predictors included psychopathy index, emotional stability, consciousness, DUP, and age. Significant negative violent offending predictors (ie, the ones that reduced the chances of belonging to the violent offending group) were extroversion, pleasantness, and intellect (Table 3).

## Discussion

Our study found that alcohol addiction was the strongest violent offending predictor, even when adjusted for other model variables. Similarly, in Scandinavia, in one of the most extensive analyses to date, it was shown that substance abuse had a strong mediating effect on the risk for violent offending in schizophrenia (1). Substance abuse is prevalent among patients with schizophrenia and is frequently established at first presentation (24). In forensic settings, it was found that 26% of patients with schizophrenia and comorbid substance abuse were violent offenders, compared to only 7% patients without substance abuse (25). The same authors showed that individuals with both schizophrenia and substance abuse were 25.2 times more likely to commit violent crimes than healthy individuals (25). Abuse has been shown to be strongly associated with treatment non-compliance, tardive dyskinesia, criminality, and suicide (26-29).

Significantly higher rates of criminal conviction and recidivism have been found in patients with a lack of insight at discharge (30). We, on the other hand, did not find significant differences between the groups in insight into the illness. One explanation for this discrepancy is that our study was conducted in illness remission. Also, using only the BCIS as self-assessment questionnaire could have further biased our results. Objective clinical assessment of DUP suggested that this indeed might have been the case. Namely, DUP was significantly longer in the violent offending group. It has been suggested that the inability to recognize illness symptoms before first treatment could be connected with mentalization inability (31,32) and that this is another probable aggression predictor in patients suffering from schizophrenia. However, researchers are still somewhat divided on this topic and some authors have not found any causal relationship between violent behavior and DUP (33), while others have reported that DUP was connected with a worse illness prognosis, increased suicide risk, and possibly serious violent behavior (34).

In agreement with other studies, we also found that patients with a higher psychopathy index score had greater chances of belonging to the violent offending group (35,36), when all other model variables were controlled, although the comparison between the two groups did not reach significance. Moreover, the violent offending group also had lower education and was more emotionally stable than non-violent offending group.

Current clinical guidelines recommend that violence risk in schizophrenia should be consistently assessed but existing approaches can be resource-intensive and detailed clinical assessments of violence risk for most patients might not always be possible, especially in economically challenged health services (2). The observed predictive factors in the Croatian sample are in agreement with other previously published studies (1-3,34,37). However, in contrast to the study by Singh et al (2), “older age” in our study was more prognostic for violence and violent offending patients were on average a decade older (43.80 years) than patients with no history of violent offending (34.83 years). Of note, there was no significant difference in age of the first contact with psychiatric services. Also, since we used the cross-sectional design, our results can only suggest an association rather than point to a clear causal relationship. In conclusion, we hope that our findings will help relegate resources toward prevention of alcohol abuse in this vulnerable patient population.
